# Book Review: Integrating Assessment Into Early Language Learning and Teaching

**DOI:** 10.3389/fpsyg.2021.700788

**Published:** 2021-06-11

**Authors:** Rodrigo A. Rodríguez-Fuentes

**Affiliations:** Foreign Languages Department, Universidad del Norte, Barranquilla, Colombia

**Keywords:** assessment and education, young learners, cognitive development, foreign language, language acquisition

After many years of independent research, the connection between cognitive development and foreign language instruction is becoming a topic of central interest among the academic community. As a result, applied linguists and young learners instructors have focused on investigating topics of the language learning process previously overlooked. One such topic is the theme of this book: the long-time call for a close relationship between assessment and emotional and cognitive development in young foreign language learners. *Integrating Assessment into Early Language Learning and Teaching* (*IAIELLAT*, henceforth) edited by Danijela Prošić-Santovac and Shelagh Rixon is focused on the issues that could establish a cause-effect relationship between affect and assessment in young learners.

The fifteen chapters of *IAIELLAT* are grouped in four parts which cast light on relevant issues regarding the interaction between cognitive and emotional factors, learning and assessment in foreign language courses for young learners, defined as 3 to 12 year-old students. Despite the singularity of its theme, the book addresses assessment dynamics from different perspectives represented in each of its four parts:

- Part 1: Why Testing is not Enough.
- Part 2: Integrating Assessment into Learning and Teaching: Approaches.
- Part 3: Integrating Assessment into Learning and Teaching: Tools.
- Part 4: From Policy to Practice Through Professional Development.

When read linearly, the book has a structure that develops from describing individual classroom experiences and case studies, through to formally structured research reports with nation-wide data. Also, *IAIELLAT* takes its empirical evidence mainly from case studies and research in Europe, a region with high demands for foreign language learning. At the end of each chapter*, IAIELLAT* summarizes the ultimate goal of each study by providing straightforward, research-based recommendations for practice.

*IAIELLAT* frequently emphasizes the relationship between the developmental constraints of cognition and foreign language abilities, which do not necessarily develop at the same pace. Indeed, lines that define knowledge of language and separate it from other emotional, cognitive and cultural biases, or contextual factors are blurred. An extremely relevant point of the book is that the assessment of young learners should include teachers' subjective opinion and bias in order to account for a focus on individual progress in the spirit of fairness. This idea of accounting for opinion and bias is opposed to norm-referenced testing and even traditional testing theory that holds bias as a threat of construct validity and, therefore, constantly seeks to reduce it (Messick, 1989; Weir, 2005).

In *IAIELLAT* Prošić-Santovac and Rixon argue that many of the principles of assessment need to be adapted, researched and discussed in relation to the unique dynamics of the pre-primary and primary classroom. Some of these adaptations require mediation of motivation, anxiety and affection according to the developmental stages that young learners experience at a certain time of their growth. Beginning with the introduction, the editors remark that it is important to bring in affective factors, and they note how these factors may change from 1 year to another, to the point of establishing affect as a curricular aim for young learners. Motivation and affection are key factors that present themselves as research opportunities in the emerging field of foreign language assessment of young learners. Likewise, Prošić-Santovac and Rixon acknowledge that the inconclusiveness of the studies carried out so far among the literature of assessment of foreign language young learners (mostly descriptive ones) is due to the myriad of measures and variables used to establish the relationships between motivation and learning, which are seldom replicable or comparable from study to study. Further complicating this research field, affective factors and language achievement are unstable and interact differently depending on the age and developmental stage of the learners, which may not be homogeneous even within the same class.

The authors and editors of *IAIELLAT* underscore throughout that in young learners, regardless of the age, assessment is likely to bring about anxiety, pressure, unease and other negative feelings that largely outweigh the positive feelings for learning another language, such as a sense of achievement and pride. Regarding this, formative assessment or assessment *for* learning (Black and Wiliam, 1998) emerges as a highly cited approach across all the chapters of the book. Assessment *for* learning is an alternative approach to evaluation that can take many forms and boasts evidence to support the claims of boosting self-confidence and motivation in young learners. To achieve effective assessment *for* learning practices, diagnostic assessment and diagnostic feedback is also required, but in the early stages of foreign language learning, self-assessment for learning is more important than learning itself, as self-assessment could determine the ownership of the learning process for the rest of the learner's academic life.

All in all, *IAIELLAT* is a necessary publication for both, psychological and second language acquisition studies as it acknowledges that, in an ideal world, there should not be tests in the pre-primary classroom or in other early stages of language learning. At the same time, the idea of not testing is in tension with arguments of accountability, information gathering, reporting and effectiveness of the learning process that are usually the reasons offered for conducting tests in formal educational settings. The alternative comes from the suggestion of less formal forms of pre-planned and incidental assessments to keep track of learners' progress. *IAIELLAT* is a collection of research-based experiences that highlight the cognitive and linguistic constructs as key factors to be considered in order to point readers in the right direction for making informed decisions regarding the use of assessment strategies for young language learners. More importantly*, IAIELLAT* opens the discussion for linguists, psychologists and researchers interested early language learning to engage in systematic research to fulfill the ambitious endeavor of creating a model of assessment of teaching foreign language in pre-primary and primary courses, a model not yet available in the field.

## Author Contributions

The author confirms being the sole contributor of this work and has approved it for publication.

## Conflict of Interest

The author declares that the research was conducted in the absence of any commercial or financial relationships that could be construed as a potential conflict of interest.
